# Integrating interventional oncology in the treatment of liver tumors

**DOI:** 10.1007/s10353-018-0521-5

**Published:** 2018-04-13

**Authors:** D. Putzer, P. Schullian, E. Braunwarth, M. Fodor, F. Primavesi, B. Cardini, T. Resch, R. Oberhuber, M. Maglione, C. Margreiter, S. Schneeberger, S. Stättner, D. Öfner-Velano, W. Jaschke, R. J. Bale

**Affiliations:** 1 Department of Radiology, Medical University Innsbruck, Anichstr. 35, 6020 Innsbruck, Austria; 2 Department of Surgery, Medical University Innsbruck, Innsbruck, Austria

**Keywords:** Radiofrequency ablation, Liver tumor treatment, Percutaneous tumor treatment, Interventional oncology, Minimally invasive oncology

## Abstract

**Background:**

Percutaneous ablation techniques offer a vast armamentarium for local, minimally invasive treatment of liver tumors, nowadays representing an established therapeutic option, which is integrated in treatment algorithms, especially for non-resectable liver tumors. The results of ablative treatment compare very well to surgical treatment in liver lesions, and confirm that these techniques are a valuable option for bridging for transplantation. Different techniques have been established to perform tumor ablation, and the feasibility varies according to the procedure and technical skills of the operator, depending on the size and location of the liver lesion. In recent years, stereotactic multi-needle techniques using 3D trajectory planning, general anesthesia, and tube disconnection during needle placement have had a strong impact on the application range of ablation for liver tumors.

**Conclusion:**

It is well known that creating a sufficient ablation margin and overlapping ablation zones is one key issue to enable ablation of large liver lesions with tumor-free margins (A0 ablation in analogy to R0 resection). Image fusion during treatment and follow-up assure highly accurate staging procedures and interventional planning.

**Novel aspects:**

Review on the standards in ablation techniques for the treatment of liver tumors. Update on different ablation techniques, indications, and contraindications for percutaneous liver tumor treatment. Summary of recently published reports on liver tumor ablation.

## Introduction

Percutaneous liver tumor ablation has developed as a minimally invasive local treatment with curative potential, and is integrated in international treatment protocols. The method has gained importance due to the fact that the technique delivers effective local therapy to many more patients than could be treated using resection alone [11, 42, 70]. The combination of a highly effective treatment and limited complication rates has been extensively described in the literature. It is subject to expert discussions where ablation fits in the management of patients with liver tumors, such as hepatocellular carcinoma (HCC), intrahepatic cholangiocellular carcinoma (ICC), or metastatic disease.

In percutaneous image-guided radiofrequency ablation (RFA), probes are inserted into the tumor using ultrasound, computed tomography, or magnetic resonance tomography [69]. The tumor is subsequently devitalized by thermal ablation applying a radiofrequency current (375–480 kHz). Microwave ablation (MWA), cryoablation, and irreversible electroporation (IRE) are alternative ablation technologies achieving local tumor destruction. However, RFA in comparison to the other ablation techniques has been extensively investigated, with studies with sufficiently large patient cohorts to produce evidence for the feasibility and effectiveness of RFA. Treatment is performed either under local or general anesthesia, depending on the technique and tumor size and location, and patients are hospitalized for 2–5 days.

When comparing surgical resection to RFA, the image-guided treatment is a low-risk procedure, while surgery is associated with a morbidity of 15–45% and mortality of 1–5% [53]. Major complications during RFA requiring intervention, such as intraperitoneal bleeding, liver abscess, intestinal perforation, pneumothorax and hemothorax, or bile duct injury, are rare incidents with a range of 2–3% and can be treated by interventional radiologists [38, 46]. The procedure-related mortality rate is below 1%. Minor complications amount to 5–9% and include the post-ablation syndrome, which is characterized by fever up to 38.5 °C, weakness, fatigue, and leukocytosis.

In a prospective randomized controlled trial comparing RFA and resection in 180 patients suffering solitary HCC with a size below 5 cm, the 1‑, 2‑, 3‑, and 4‑year survival was 95.8%, 82.1%, 71.4%, and 67.9%, respectively, and after resection 93.3%, 82.3%, 73.4%, and 64%, respectively [12]. RFA is less invasive, resulting in lower complication rates and lower overall treatment costs. Comparison of patient cohorts treated either by resection or RFA is hampered when patients not suitable for resection are treated by locally ablative treatment approaches [39]. When comparing RFA to transarterial chemoembolization (TACE), TACE produces response rates between 17 and 62%, with a low rate of complete responses (0–5%) [59]. Therefore, TACE is not regarded as a locally curative treatment approach [67].

The feasibility and success of RFA depend on the size and location of the liver lesion. RFA is contraindicated in the case of insufficient liver remnant, vicinity of tumor to the central bile duct of the liver, presence of a biliodigestive anastomosis. Lesions with a diameter larger than 1 cm usually require more than one probe or several probe positions in order to treat the tumor with overlapping ablation zones. These can result ellipsoidal, using single straight electrodes, or spherical, applying single expandable electrodes. Incomplete RFA leading to local recurrence is mostly associated with large size tumors (>3–5 cm), poor tumor visibility, and unfavorable distribution of probes, as well as imprecise probe positioning and cooling effects by larger vessels, which has been attributed the heat sink effect. The technical limitations of conventional single-needle in-plane techniques using US or CT have been largely overcome by multi-needle approaches using CT-based 3D treatment planning and stereotactic needle guidance [4, 5]. The Innsbruck approach to RFA includes the planning of overlapping ablation zones using 3D data sets to increase the spectrum of locally curable liver lesions [3–5]. The so-called “stereotactic radiofrequency ablation (SRFA)” allows effective and safe treatment of large-volume disease [6]. In analogy to surgical R0 resection, A0 ablation including a 3D safety margin of at least 5 mm can be objectively verified and documented by fusion of post-ablation and pre-ablation contrast-enhanced images [18]. Use of multimodal fused images from PET-CT during SRFA may permit selective treatment of active metastasis as determined by tracer uptake [63].

The technical feasibility in RFA depends on the anatomical tumor location more than on tumor size, according to the technique applied. This makes referral of technically demanding interventions to specialized centers an essential point. Feasibility of RFA should be discussed in interdisciplinary oncologic boards.

## Hepatocellular carcinoma

The incidence of HCC is increasing worldwide in correlation with obesity and liver steatosis [61]. The 5‑year survival of HCC patients is poor, with about 10–15% [48], as patients are often diagnosed in an advanced stage of disease. Advances in percutaneous treatment approaches have led to the development of efficacious ablation techniques for curative treatment. A vast range of percutaneous ablation techniques are available to treat HCC, including monopolar, RFA, bipolar RFA, MWA, cryoablation, and IRE.

### Radiofrequency ablation

According to international guidelines, such as EASL-EORTC clinical practice guidelines, RFA is one of the main curative treatments of HCC. In the AASLD guidelines, smaller single tumors with diameters below 2.5 cm may be equally well treated by either resection or conventional ultrasound- or CT-guided ablation [20]. The ablative approach has been studied extensively in cirrhotic patients, in combination with surgery and liver transplantation. Ablation can be used as bridging therapy to liver transplantation, or sequentially [17]. Further innovations in ablation techniques allow extension of ablation criteria beyond early HCC. The use of multiple bipolar RFA probes with a no-touch technique also allows treatment of tumors in difficult to reach locations [26].

The application of an electric current through ablation probes in the tumor allows application of temperatures of 60–100 °C, leading to coagulation necrosis. In our center, SRFA is performed in patients under general anesthesia and muscular blockade, using tube disconnection during the planning phase, needle advancement, and control CT scans, to allow for precise needle placement and image registration of pre- and postoperative image data sets. The size of the necrosis depends on the distribution of heat from the electrode tip to the periphery of the tumor, as well as on the blood flow at the site of ablation, leading to the so-called heat sink effect. This effect can be overcome by increased duration and power of ablation, with ablation probes preferentially positioned in the region of the tumor next to the vessel site. RFA is the most widely used ablation technique, allowing for accurate local tumor control, and meta-analysis has proven that RFA improves the overall survival [48], making it the standard ablation technique, which can be combined with surgical resection. Different randomized controlled trials have proven its superiority over other percutaneous treatment approaches [10, 32, 35, 36, 66]. The use of stereotactic navigation tools and image fusion during SRFA procedures increases the treatable volume and also predictability of treatment results [5]. Recently published papers have proven the effectiveness of RFA in HCC larger than 5 cm, and in patients with Barcelona Clinic Liver Cancer (BCLC) stage 2 [2, 37].

Even in studies including patients with multifocal HCC, RFA and MWA showed satisfactory long-term results. Treatment combination of RFA and TACE may improve treatment outcomes in advanced stage HCC, and prospective randomized trials are mandatory to further evaluate the combination therapy [34].

In RFA studies on HCC patients with cirrhotic liver, major complications have been described in 1–5% of patients, with a mortality as low as 0.3% [25, 40, 49, 50, 60]. Randomized controlled trials have confirmed that side effects and mortality for patients who are treated by RFA are significantly lower than for HCC patients undergoing surgical resection [16]. Perioperative complications in RFA of HCC include a post-ablation syndrome, characterized by pain and fever. Possible complications include pleural effusion, pneumothorax, bleeding, hemoperitoneum, liver failure, abscess, bilioma, perforation of the gastrointestinal tract, tumor seeding, and thermal injury of the skin. The size of the ablation zone and the underlying liver function strongly influence the risk for complications. Therefore, tumors located right next to the central bile duct are a contraindication for RFA and should be treated by IRE instead. A history of bilioentereic anastomosis or the presence of aerobilia are contraindications for ablative procedures, as there is a high risk of abscess formation within the coagulation necrosis deriving from bile duct colonization and cholangitis. High-flow bile duct cooling could be performed in tumors located next to a central bile duct; however, there is no report in the literature to confirm the effectiveness. Relative contraindications such as thrombocytopenia can be overcome by platelet transfusion or application of thrombopoiesis-stimulating agents.

In order to obtain a complete radiological response, which is associated with prolonged overall survival [30, 62], multipolar RFA is the method of choice, and has shown advantages over monopolar approaches [41, 44]. In case of early or late tumor recurrence, which is defined by the appearance of new tumor sites in less or more than 2 years after treatment, respectively, RFA can efficiently treat the tumor, maintaining a low complication rate and restricting postoperative hospitalization to a minimum. Recurrence of HCC can be caused by an insufficient ablation margin, aggressive tumor biology, and the so-called heat sink effect in HCC located next to vessels. According to recently published literature, an ablation margin of 0.5–1 cm is sufficient to overcome these limitations [21, 47].

Different studies have shown that results of RFA compare very well to surgical results, while further reducing postinterventional morbidity and mortality and costs [13, 14, 24, 31, 56]. However, the feasibility of RFA in patients who are not resectable or suffer significant comorbidities led to selection of patients with advanced and severe liver disease in the RFA treatment group, hampering the direct comparison of RFA and surgery [57].

In contrast to TACE, RFA is a potentially curative treatment approach, which can also be combined with liver transplantation after RFA as first-line treatment [29]. Prognostic factors such as tissue biomarkers including AFP, DCP, and VEGF have to be taken into consideration when planning treatment, as these markers correlate with an increased risk of tumor recurrence when increased [58, 71].

RFA even offers the possibility of percutaneous treatment of HCC metastases to the lungs or lymph nodes, if clinically feasible [19, 33, 54]. However, there are not sufficient data to validate the treatment of angioinvasive HCC.

### Microwave ablation

MWA is based on the principle of heat induction through creation of an electromagnetic current around a monopolar electrode, leading to coagulation necrosis. This technique allows higher temperatures to be reached faster than RFA, shortening the time needed to treat the tumor. Up to now, MWA has shown comparable therapeutic results to RFA of HCC in clinical studies [52]. The technique has also shown comparable results to RFA regarding outcome and complication rate in HCC treatment [1, 65].

### Irreversible electroporation

This innovative ablation technique is not based on thermocoagulation, but on induction of apoptosis by application of short electric high-frequency pulses in between two electrodes. This leads to the disruption of the cell membrane. The procedure requires general anesthesia and muscular blockade, and is then performed in synchrony with the heartbeat to prevent cardiac arrhythmia. The technique allows treatment of HCC situated close to vessels and biliary structures, which cannot be treated by RFA. Up to now, the literature on IRE is restricted to evaluations in small HCC patient cohorts [15]. However, long-term results are not yet available.

### Cryoablation

The application of ablation probes using argon or helium gas to freeze the tissue by creation of a defined ice ball potentially has the advantage of direct monitorization of the treatment effect by visualization of the ice ball surrounding the ablation probe. Different cryoablation probes are available, creating predefined ice balls, allowing prediction of the size of the ice ball necessary to cover the target lesion. However, the first studies on cryoablation reported an increasing number of adverse events compared to other ablation techniques [23, 55]. Different approaches have been described to precisely position the cryoablation probes, including MRI guidance [43]. Furthermore, the cyroprobes do not generally allow needle tract ablation, which is mandatory to prevent tumor seeding.

## Cholangiocellular carcinoma

For patients with advanced, inoperable intrahepatic cholangiocellular carcinoma, there is no standardized curative treatment regimen available. Up to now, only resection and liver transplantation are considered to be curative options. Systemic chemotherapy is available. However, alternative locoregional therapy results are based on small, single-center reports, including RFA, MWA, TACE, selective internal radiation therapy (SIRT).

**Fig. 1:**
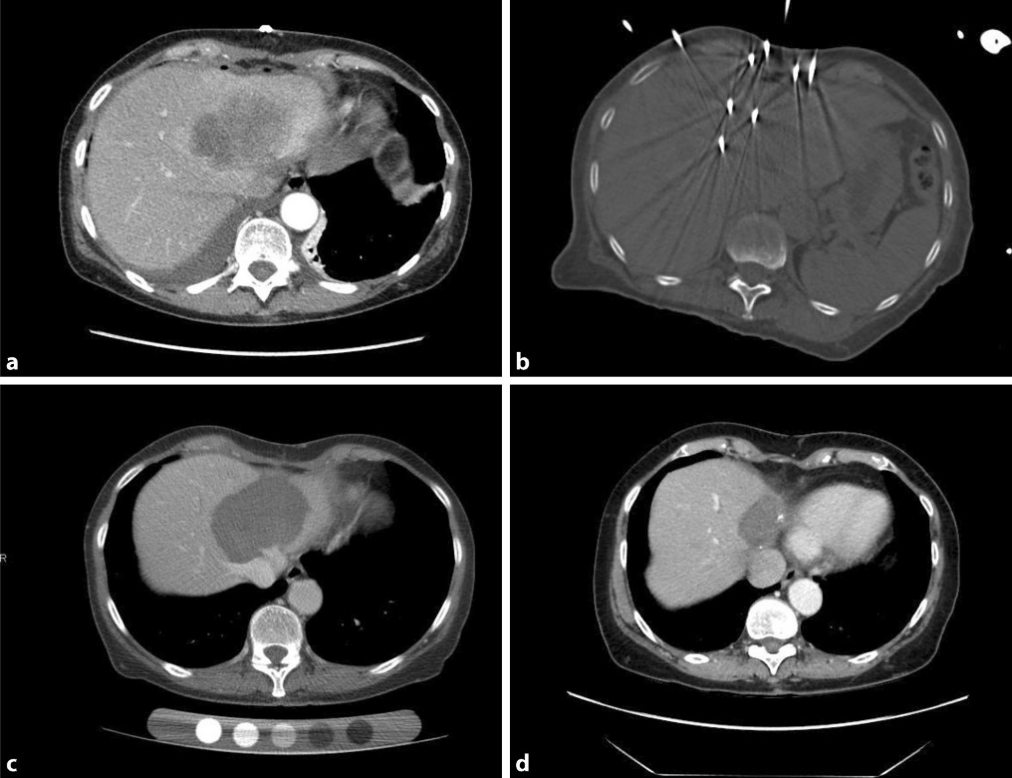
63-year-old female patient with inoperable intrahepatic cholangiocellular carcinoma (ICC) measuring 10 cm in diameter diagnosed in 2010 (**a**). Patient underwent radiofrequency ablation (RFA) 02/2010 using 12 coaxial needles (**b**). Follow-up 05/2010 showed complete ablation (**c**). Follow-up in 2017 showed no sign of recurrence (**d**)

SRFA is a promising ablation technique, allowing precise 3D planning and exact positioning of multiple probes in difficult to reach locations (Fig. 1). Larger tumors can be treated maintaining a safety margin, assuring A0 ablation and showing a clear survival benefit compared to other palliative treatment options [18]. Our group has treated 17 inoperable ICC patients with 52 tumors by SRFA, and patients reached a median overall survival of 60 months postoperatively. Even large tumors with more than 10 cm diameter were ablated, which marks a clear improvement in comparison to the other techniques available [7]. Our results were confirmed by reports from other working groups showing comparable results in smaller patient cohorts, reaching a median local progression-free survival and overall survival of 32.2 and 38.5 months, respectively [27]. This makes SRFA an attractive minimally invasive approach for ICC treatment in the first-line setting.

## Metastases

**Fig. 2:**
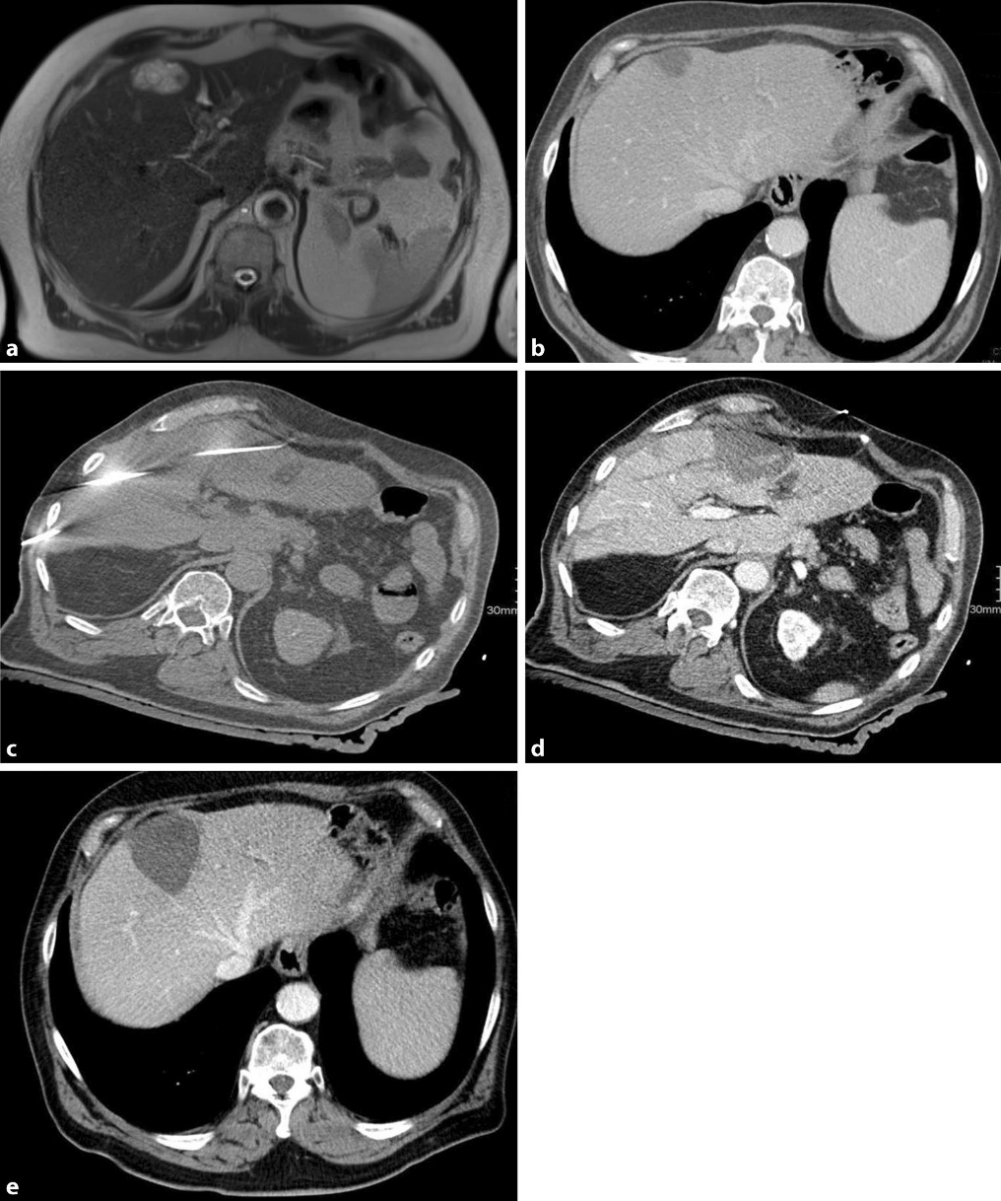
72-year-old male patient. First diagnosis of an moderately differentiated adenocarcinoma of the stomach, grade II, TNM pT3N1(1/26)L1V0R0, UICC stage IIIA. Patient underwent gastrectomy in 2012. MRI Follow up on 21/07/2015 showed solitary liver metastasis, which was histologically confirmed (**a**). SRFA on 11/12/2015 (**b**). Needle placement (**c**). Postinterventional control CT, showing sufficient ablation margin (**d**). Follow-up 7/09/2016, with no sign of recurrent disease (**e**)

In accordance with the results of ablative treatment of primary liver tumors, several studies have shown the clinical efficiency of percutaneous local ablative treatment approaches in patients with liver metastases. Local treatment is generally applicable in oligometastatic disease, depending on primary tumor location and tumor diameter (Fig. 2).

A recently published study reported the value of RFA in the treatment of oligometastatic pancreatic cancer with synchronous liver metastases in 102 patients, in accordance with NCCN guidelines [22]. The authors report a 1-year survival rate of 47.1% and a median overall survival of 11.4 months. Complete tumor ablation was achieved in 94.5% of all interventions, and no severe complication was witnessed.

Our group has produced evidence to suggest that large colorectal liver metastases can be effectively treated by SRFA, directly influencing overall survival in a patient cohort of 63 individuals who underwent 98 SRFA sessions for ablation of 189 colorectal liver metastases [6]. The median overall survival was 33.2 months, and the corresponding 1‑, 3‑, and 5‑year survival rates were 87%, 44%, and 27%, respectively. In a subgroup analysis, focusing on patients with resectable colorectal liver metastases, the overall survival rate was significantly higher, reaching 92%, 66%, and 48% for 1‑, 3‑, and 5‑year survival rates, respectively. Interestingly, tumor size was not predictive for treatment outcome. SRFA could be applied as first-line local treatment according to these results; however – as for hepatic resection –, no prospective, randomized trial is yet available to confirm these results.

In accordance with our findings, a recently published study reports on the effectiveness of thermal ablation techniques for treatment of colorectal liver metastases [64]. The authors state that local tumor control can be effectively reached by maintaining an ablation margin of more than 5 mm. No local tumor progression was noted for ablation zones with a safety margin larger than 10 mm. In this retrospective study, 110 patients were included. No significant differences in local tumor progression rate were observed between RFA and MWA. In multivariate analysis, radiofrequency ablation margins less than 5 mm and perivascular tumor localization were significant predictors of shorter time to tumor progression. Additional tumors developing during follow-up should be considered for further ablation to maintain tumor control using a minimally invasive therapeutic approach with low complication rates.

Recently, our study group has published data on the outcomes of patients with liver metastases from breast cancer treated by SRFA, involving 29 treatment sessions in 26 patients with 64 histologically confirmed liver metastases from breast cancer [8]. Patients had not responded to systemic treatment. Primary and secondary technical success rates were 96.9% and 100%, respectively, without any major complications. With a median follow-up of 23.1 months, the local recurrence rate was 7.8%, and the median estimated overall survival from first SRFA was 29.3 months. Overall survival rates were not influenced by tumor volume or number of metastases. SRFA can be used as a minimally invasive alternative to surgical resection in selected breast cancer patients.

RFA has proven efficacy in treatment of neuroendocrine tumor liver metastases. A systematic review including 8 studies with 301 patients reports on a 92% symptom improvement following RFA, with a median duration of 14–27 months [45]. Tumor recurrence was observed in 63–87%, indicating the need for combination treatment including local ablative and systemic treatment approaches.

## Outlook

Retrospective studies have produced evidence supporting the hypothesis that combination of local treatment approaches such as RFA and TACE allows effective treatment of tumors that cannot be easily overcome by a single technique, especially in larger tumors with unfavorable tumor biology [28, 68]. These studies indicate the potential to reduce recurrence rates and improve overall survival. However, no randomized controlled trial is available up to now.

A potential benefit of percutaneous local ablative treatment and adjuvant systemic treatment has not yet been confirmed. One placebo-controlled trial comparing sorafenib treatment in 1114 patients after surgery or RFA did not result in any significant improvement of overall survival compared to sorafenib therapy alone [9]. Immunotherapy still carries the potential to booster immune response mechanisms that are already triggered by ablation-induced coagulation necrosis. However, to date, reports in the literature are based on findings of RFA in a tumor-bearing mouse model [51].

## Conclusion

Percutaneous ablation techniques offer a wide armamentarium to treat and control oncologic liver disease. Stereotactic navigation and image registration techniques allow safe treatment of liver lesions with diameters larger than 5 cms in a curative setting. A multidisciplinary treatment approach has the potential to further improve oncologic liver treatment.
